# Gynaecological Morbidities and Treatment-Seeking Behaviour Among Post-menopausal Women in Kerala: A Cross-Sectional Study

**DOI:** 10.7759/cureus.93474

**Published:** 2025-09-29

**Authors:** Thilak K Theertha, P Sankara Sarma, Jeby J Olickal, Kavumpurathu R Thankappan

**Affiliations:** 1 Department of Public Health, Amrita School of Medicine, Amrita Institute of Medical Sciences, Amrita Vishwa Vidyapeetham, Kochi, IND

**Keywords:** gynaecological morbidity, india, influencing factors, postmenopausal women, prevalence

## Abstract

Background

Gynaecological morbidities (GMs), including pelvic organ prolapse, urogenital infections or inflammations, benign and malignant genital tract disorders, and postmenopausal bleeding, are significant but under-recognised health concerns among postmenopausal women. Limited data are available on their prevalence and treatment-seeking behaviour in Kerala. This study aimed to estimate the prevalence of GMs, identify associated factors, and assess treatment-seeking patterns.

Methods

A community-based cross-sectional study was conducted in Kannur and Ernakulam districts of Kerala, representing the northern and southern regions of the state. Using multi-stage cluster sampling, 384 postmenopausal women aged 45-69 years were selected from 24 clusters. Data were collected through a pre-tested, semi-structured interview schedule covering sociodemographic characteristics, self-reported GMs, and healthcare access. Descriptive statistics were used to estimate prevalence and treatment patterns, chi-square tests assessed associations, and multivariable logistic regression identified factors independently associated with GMs. Treatment-seeking was defined as consultation with a healthcare provider, while receipt of treatment indicated initiation of medical care.

Results

The prevalence of GMs was 14.1% (n = 54, 95% CI: 10.9-17.8). The most commonly reported conditions were urogenital infections or inflammations (5.0%), benign genital tract disorders (5.5%), pelvic organ prolapse (2.8%), malignant genital tract disorders (0.5%), and postmenopausal bleeding (0.3%). Higher odds of GMs were observed among women aged 45-59 years (adjusted odds ratio (AOR) = 2.13, 95% CI: 1.01-4.46), those with diabetes mellitus (AOR = 2.99, 95% CI: 1.44-6.21), thyroid disorders (AOR = 4.21, 95% CI: 2.08-8.52), and early menopause (AOR = 2.20, 95% CI: 1.15-4.21). All affected women sought consultation; however, only 63.6% received treatment. Most approached private hospitals (74.1%) and gynaecologists (66.7%).

Conclusion

Approximately one in seven postmenopausal women reported GMs. Despite universal consultation, treatment uptake was suboptimal. Strengthening public health services, increasing awareness, and addressing barriers to care are essential to improve outcomes for this population.

## Introduction

Gynaecological morbidity (GM) refers to any condition, disease, or dysfunction of the reproductive system that is unrelated to pregnancy, abortion, or childbirth, although it may be associated with sexual behaviour [1]. It encompasses reproductive tract infections, cervical cell abnormalities, pelvic organ prolapses, infertility, and urinary tract infections [2]. Gynaecological conditions are significant contributors to morbidity and mortality worldwide, disproportionately affecting women in low-resource countries (LRCs). These conditions account for approximately 4.5% of the global disease burden, surpassing malaria (1.0%), tuberculosis (1.9%), ischaemic heart disease (2.2%), and maternal health conditions (3.5%) [3].

The prevalence of GM in community-based studies ranges from 43% to 92% among women of all age groups in the general population. These variations are influenced by sociocultural, demographic, and behavioural factors [4]. Several studies have highlighted the high prevalence of GM among postmenopausal women globally. However, overall prevalence is often underreported, as most research focuses on individual conditions [4]. A recent nationally representative study in India investigated GM and treatment-seeking behaviour among older adult women aged 45-59 years, using data from 18,547 participants. The study found that 15% of women reported experiencing at least one form of GMs, including symptoms such as vaginal bleeding, foul-smelling discharge, uterine prolapse, and vaginal dryness. However, only 41% of these women sought medical treatment. Various sociodemographic factors were significantly associated with both the prevalence of GM and health-seeking behaviour, including age, marital status, educational attainment, parity, hysterectomy status, decision-making power within the household, caste, religion, economic status, and regional disparities [5].

In India, the population aged 60 years and above was about 104 million according to the 2011 Census and is projected to rise to nearly 319 million by 2050. Among them, more than 52 million are women, a substantial proportion of whom live in rural settings with limited access to specialised healthcare services [6]. The average life expectancy of Indian women has increased to 71 years, with 68.7 years in rural areas and 73.5 years in urban areas [7]. As a woman approaches menopause, her hormone levels begin to change [8]. Menopause is a major milestone in every woman's life, accompanied by notable physiological changes [9]. Natural menopause refers to the permanent cessation of menstruation due to the natural decline in ovarian follicular activity. It is considered to have occurred after 12 consecutive months of amenorrhea, provided there is no other clear pathological or physiological reason for the absence of menstruation. The natural onset of menopause typically occurs between 40 and 55 years of age globally [10]. Menopause represents a critical phase in a woman’s life, historically overlooked or concealed. It is now widely acknowledged as a challenging period, with around 80% of women experiencing physical and psychological disorders during this stage [11]. Evidence suggests that women often do not seek medical care, as they perceive the physical discomfort associated with gynaecological issues, menopause, and ageing as natural and inevitable [12]. Many women attribute the health concerns of this life stage to ageing rather than menopause itself [13]. In Indian society, women commonly place greater emphasis on the health of their children and family members than on their own, resulting in the frequent neglect of women’s health needs, especially gynaecological concerns [14]. In certain societies, cultural norms and stigma lead women to avoid seeking professional help for gynaecological issues due to embarrassment, shame, or the belief that such conditions are normal [15].

Existing research on postmenopausal women in Kerala is limited, often small-scale, facility-based, or focused only on menopausal symptoms, leading to inconsistent prevalence estimates and insufficient understanding of treatment-seeking behaviours. A community-based study conducted in an urban setting in the Trivandrum district assessed the prevalence and pattern of direct GM among postmenopausal women aged over 50 years. It found that 19% reported gynaecological symptoms, while morbidity confirmed through clinical examination was 23.5%, with urinary tract symptoms being the most common. Lower socioeconomic status and younger age at menarche were significantly associated with gynaecological symptoms [16]. Despite the significant burden of conditions ranging from reproductive tract to urinary tract infections, current research is insufficient in capturing their prevalence and management. Therefore, this study aimed to determine the prevalence of GM, identify the factors associated with them, and assess patterns of treatment-seeking behaviour.

## Materials and methods

Study design

This was a community-based observational study.

Study setting

The study was conducted between August 2024 and May 2025 in two districts of Kerala, Kannur in the north and Ernakulam in the south, to ensure regional representation.

Study population

The study population comprised postmenopausal women aged 45-69 years residing in the selected districts of Kerala.

Postmenopausal women aged 45-69 years were included, while those with cognitive impairment were excluded from the study.

Sample size

The present study assumed a 40% prevalence of GM among women aged 45-69 years, based on previous evidence of high morbidity levels in similar populations [4]. Using an absolute precision of 7%, a 95% confidence level, and a design effect of 2, the required sample size was calculated to be 377 using OpenEpi Version 3.0 (Dean AG, Sullivan KM, Soe MM, Emory University, Atlanta, GA). To ensure adequate representation, the sample size was rounded up to 384, with 16 participants selected from each of the 24 clusters.

Sampling method

**Figure 1:**
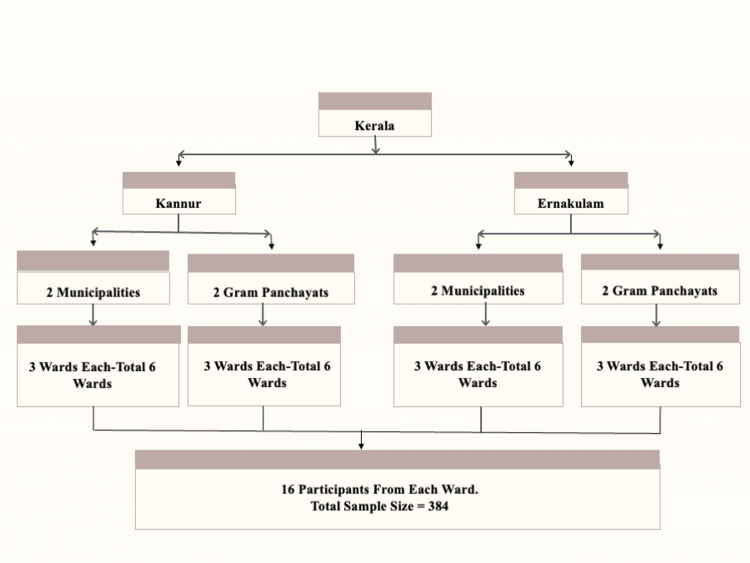
Sampling method

A multi-stage cluster sampling method was used. From the 14 districts of Kerala, one district each from the northern and southern regions was randomly selected. Within each selected district, two municipalities and 2 g panchayats (the lowest tier of rural local government in India, consisting of elected village councils responsible for administration and delivery of basic services) were randomly chosen to represent urban and rural populations. From each municipality and gram panchayat, three wards were randomly selected, each ward serving as a cluster, resulting in a total of 24 clusters (12 from each district) (Figure 1).

The central location of each ward was identified, and a random direction was chosen. House-to-house visits were then conducted in the selected direction until 16 eligible participants were enrolled. After obtaining written informed consent, data were collected using the study tool. A total of 384 participants were interviewed - 16 from each of the 24 clusters.

Study duration

The study spanned nine months, from August 2024 to May 2025. Data collection was carried out from 17 December 2024 to 17 February 2025.

Study tool and administration process

A pre-tested semi-structured interview schedule was used for data collection. It consisted of three sections: Section I: Socio-demographic and behavioural characteristics. Section II: Gynaecological factors, including variables such as age at menarche (in years), parity, and age at menopause (in years), as well as symptoms and morbidities, were collected. Section III: Treatment-seeking behaviour related to gynaecological symptoms and morbidities.

The tool was translated into Malayalam and back-translated by experts to ensure consistency. Data collection was carried out through house-to-house interviews.

Validation of the tool

The semi-structured interview schedule underwent content and face validation by experts in public health and gynaecology. The English tool was translated into Malayalam and back-translated by independent bilingual experts; discrepancies were reconciled to ensure conceptual equivalence. The tool was pre-tested among a small sample (n = 10) of postmenopausal women from a non-study area to assess clarity and flow, and minor revisions were made. 

Data collection and quality control

Data were collected by a trained postgraduate student researcher. Prior to data collection, the interviewer underwent comprehensive training, which included familiarisation with the study tool, role-playing interviews, and clarification of variable definitions to ensure uniform understanding. Standardised procedures were followed during all house-to-house interviews to minimise interviewer-related variability.

Reliability of administration was ensured through interviewer training and the use of standard operational definitions. The data collection process was supervised, and entries were periodically reviewed by the research guide to ensure accuracy and consistency. To address potential self-report bias, including recall and social desirability bias, measures were implemented, such as ensuring participant privacy, asking neutral and non-leading questions, and clarifying responses during the interview.

Study variables

Independent Variables 

The independent variables included both socio-demographic and reproductive factors. Age was categorised into two groups: 45-59 years and ≥60 years. Marital status was classified as currently married or others (including widowed, separated, or never married). The presence of diabetes mellitus and thyroid disorders was assessed based on self-report of a prior medical diagnosis and was coded as yes or no. Age at menarche was dichotomised as <14 years and ≥14 years, while age at menopause was classified as <47 years and ≥47 years, based on the median age observed in the study population and consistent with previous literature.

Outcome Variable

The primary outcome variable was the presence of any GM, defined as reporting at least one of the following conditions during the interview: pelvic organ prolapse, malignant disorders of the genital tract, urogenital infections or inflammations, benign disorders of the genital tract (e.g., fibroids, ovarian cysts), or postmenopausal bleeding (PMB).

For analysis, the prevalence of each individual morbidity was calculated separately, and a composite variable “any GM” was created to represent women reporting one or more of the above conditions.

Data analysis

Data were entered in Microsoft Excel (Microsoft® Corp., Redmond, WA) and analysed using jamovi version 2.3.28 (Jonathon Love, Damian Dropmann, and Ravi Selker, Sydney, Australia) and IBM SPSS Statistics for Windows version 20 (IBM Corp., Armonk, NY). Categorical variables were summarised as frequencies and percentages. The prevalence of GM, as well as of individual conditions, was estimated with corresponding 95% confidence intervals (CIs). Treatment-seeking patterns were described using descriptive statistics, including the proportion of women who sought treatment, the type of healthcare provider consulted, and the source of care utilised.

The association between the presence of any GM (primary outcome) and independent variables was examined using binary logistic regression. Unadjusted odds ratios (UORs) with 95% CIs were first estimated in bivariate analyses. Variables with a p-value < 0.20 in bivariate analysis were included in the multivariable logistic regression model to identify factors independently associated with GM. Adjusted odds ratios (AORs) with 95% CIs were reported, and a p-value < 0.05 was considered statistically significant.

Ethical considerations

The study protocol was reviewed and approved by the Institutional Ethics Committee (ECASM-AIMS-2024-593). Written informed consent was taken from all the participants. For participants who were illiterate, the consent form was read aloud in their presence, and a thumb impression was obtained in lieu of a signature. For differently abled participants, appropriate assistance was provided to ensure they fully understood the study purpose, procedures, and their rights before providing consent. Participants were assured of confidentiality, voluntary participation, and their right to withdraw at any time without any consequences.

## Results

A total of 384 postmenopausal women participated in the study. The majority were in the 45-59-year age group (n = 231, 60.2%) and most were currently married (n = 313, 81.5%). Regarding socioeconomic and demographic characteristics, 141 (36.7%) belonged to the BPL category and 243 (63.3%) to the APL category, 192 (50.0%) resided in urban areas and 192 (50.0%) in rural areas, and 155 (40.4%) had health insurance coverage. Most participants had attained either primary (n = 149, 38.8%) or secondary education (n = 157, 40.9%) and followed a predominantly mixed diet (n = 362, 94.3%). Inadequate physical activity was reported by 325 (84.6%) women. The most commonly reported chronic conditions were hypertension (n = 116, 30.2%), diabetes (n = 76, 19.8%), and thyroid disorders (n = 57, 14.8%) (Table 1).

**Table 1:** Sociodemographic and behavioural characteristics of the study participants (n = 384)

Variables	n	%
Sociodemographic characteristics		
Age (in years)		
45-59	231	60.2
≥60	153	39.8
Education		
Primary level	149	38.8
Secondary level	157	40.9
Higher secondary and above	78	20.3
Marital status		
Currently married	313	81.5
Others	71	18.5
Socio economic status		
BPL	141	36.7
APL	243	63.3
Residence		
Urban	192	50.0
Rural	192	50.0
Health insurance coverage	155	40.4
Behavioural characteristics		
Diet		
Vegetarian	22	5.7
Mixed	362	94.3
Physical activity (moderate to vigorous)		
Inadequate	325	84.6
Adequate	59	15.4
Chronic diseases		
Hypertension	116	30.2
Diabetes	76	19.8
Dyslipidaemia (high cholesterol)	68	17.7
Thyroid disorders	57	14.8
Others	6	1.6
Gynaecological factors		
Age at menarche (in years)		
<14	150	39.1
≥14	234	60.9
Parity		
≤2	212	55.2
>2	172	44.8
Age at menopause (in years)		
<47	124	32.3
≥47	260	67.7

**Figure 2:**
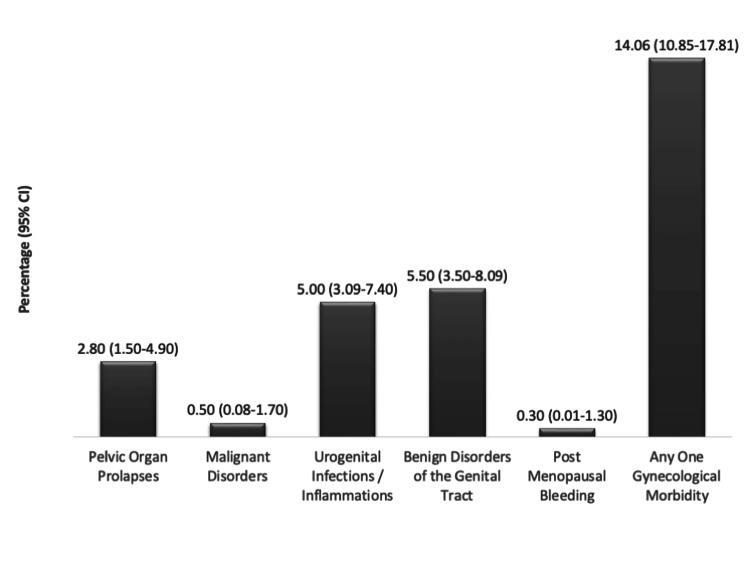
Prevalence of gynaecological morbidities among post-menopausal women in Kerala

The prevalence of GM was 14.1% (n = 54, 95% CI: 10.85-17.81) (Figure 2).

Among those with GM, the highest prevalence was observed in the 45-59 year age group (n = 41, 17.8%), with significantly greater odds compared to those aged ≥60 years (adjusted odds ratio (AOR) = 2.13, 95% CI: 1.01-4.46; p = 0.045). Significant associations were also found between GM and self-reported diabetes (AOR = 2.99, 95% CI: 1.44-6.21; p = 0.003), thyroid disorders (AOR = 4.21, 95% CI: 2.08-8.52; p < 0.001), and early menopause before the age of 47 years (AOR = 2.20, 95% CI: 1.15-4.21; p = 0.017). Other variables, such as age at menarche and marital status, were associated with increased odds of GM but did not reach statistical significance (Table 2).

**Table 2:** Factors associated with gynaecological morbidities among post-menopausal women in Kerala: results from bivariate and multivariable logistic regression analysis (n = 384) AOR - adjusted odds ratio, CI - confidence interval, UOR - unadjusted odds ratio

Variables	Gynaecological morbidity		UOR (95% CI)	p-value	AOR (95% CI)	p-value
	(Yes) n (%)	(No) n (%)				
Age (in years)						
45-59	41 (17.8)	190 (82.2)	2.32 (1.20-4.50)	0.012	2.13 (1.01-4.46)	0.045
≥60	13 (8.5)	140 (91.5)	1		1	
Marital status						
Currently married	49 (15.6)	264 (84.4)	2.45 (0.93-6.39)	0.067	1.93 (0.67-5.51)	0.221
Others	5 (7.0)	66 (93.0)	1		1	
Diabetes						
Yes	16 (21.1)	60 (78.9)	1.89 (0.99- 3.62)	0.053	2.99 (1.44-6.21)	0.003
No	38 (12.3)	270 (87.7)	1		1	
Thyroid disorders						
Yes	18 (31.6)	39 (68.4)	3.73 (1.93-7.19)	<0.001	4.21 (2.08-8.52)	<0.001
No	36 (11.0)	291 (89.0)	1		1	
Age at menarche (in years)						
<14	27 (18.0)	123 (82.0)	1.68 (0.94-3.00)	0.078	1.81 (0.97-3.39)	0.062
≥14	27 (11.5)	207 (88.5)	1		1	
Age at menopause (in years)						
<47	24 (19.4)	100 (80.6)	1.84 (1.02-3.30)	0.041	2.20 (1.15-4.21)	0.017
≥47	30 (11.5)	230 (88.5)	1		1	

All 54 women identified with GMs reported seeking medical consultation specifically for their condition (n = 54, 100%). However, only 35 (63.6%) went on to receive treatment. A total of 19 participants did not receive treatment: seven with pelvic organ prolapses, seven with urogenital infection/inflammation, and five with benign disorders of the genital tract. The majority sought care from private healthcare facilities (n = 40, 74.1%) and consulted gynaecologists (n = 36, 66.7%). Twelve women (21.8%) approached government hospitals, while one (1.8%) relied on traditional medicine practitioners. General practitioners were consulted by 12 women (21.8%), Ayurvedic doctors by four (7.3%), and others (homoeopathic doctors and traditional medical practitioners) by two (3.6%). Five women in the study reported financial constraints (9.0%) (Table 3). Of the total study population, 155 participants (40.4%) had health insurance coverage.

**Table 3:** Treatment-seeking behaviour of the participants (n = 54)

Variables	n	%
Consulted doctor	54	100
Received treatment	35	63.6
Medical facility approached		
Government hospital	13	24.1
Private hospital/clinic	40	74.0
Traditional medicine practitioner	1	1.9
Type of healthcare provider		
General practitioner	12	22.2
Gynaecologist	36	66.7
Ayurvedic doctor	4	7.4
Others	2	3.7
Any financial constraints	5	9.0

## Discussion

This community-based study aimed to estimate the prevalence of GM and examine treatment-seeking behaviour among postmenopausal women in Kerala. The overall prevalence of GM was 14.1%, comprising structural disorders such as pelvic organ prolapse (2.8%); tumours, including malignant genital tract disorders (0.5%) and benign genital tract disorders (5.5%); infections or inflammations of the urogenital tract (5.0%); and functional disorders such as PMB (0.3%). Although all women with GM consulted a healthcare provider, only about two-thirds received treatment, indicating potential gaps in the continuum of care. 

The observed prevalence of GM in this study was lower than global estimates. For instance, the global prevalence of pelvic organ prolapse among women over the age of 40, particularly postmenopausal women, is estimated to range from 41% to 50%, with approximately 40% of women worldwide expected to experience the condition during their lifetime [17]. In contrast, the prevalence reported in this study was considerably lower at 2.8%. Similarly, the prevalence of malignant genital disorders (0.5%) was lower than the global data. In 2020, cervical cancer alone accounted for 604,127 new cases globally, with postmenopausal women contributing significantly to this burden [18]. Specifically, in 2019, approximately 879,476 postmenopausal women worldwide were diagnosed with gynaecological malignancies, with cervical cancer being the most common subtype in low- and middle-income countries (LMICs) [19]. The lower prevalence of malignant genital disorders in this study may partly reflect under-detection in community-based surveys and methodological differences compared to facility-based or clinically validated studies.

The 5.5% prevalence of benign genital disorders identified here aligns more closely with international findings. Benign gynaecological conditions (BGCs), including uterine fibroids, ovarian cysts, and endometrial polyps, contribute significantly to the global disease burden, accounting for 5.35% of all years lived with disability (YLDs) in LMICs [3]. Among these, uterine fibroids represent a major subset, affecting 20% to 77% of women during their lifetime, with an estimated prevalence of 40-60% among adult women worldwide [20]. PMB, observed in only 0.3% of participants, is relatively rare and consistent with the literature indicating that structural or malignant causes are the predominant contributors [21]. A mixed-methods study among rural women in Tamil Nadu, India, similarly reported a PMB prevalence of only 1.8% [22], further underscoring the low occurrence of PMB in community-based samples. These findings reinforce that dysfunctional bleeding is uncommon in postmenopausal women and that most cases of bleeding warrant evaluation for structural or malignant causes.

At the national level, the 14.06% prevalence of GM reported in this study is comparable to findings from the Longitudinal Ageing Study in India (LASI) 2016-2017, which documented a prevalence of approximately 15% among women aged 45-59 years [5]. Differences across studies may be explained by variation in study design, diagnostic criteria, and healthcare access, particularly the greater likelihood of community-based surveys to detect different morbidity patterns compared to facility-based assessments [23]. The 0.3% prevalence of PMB reported here contrasts sharply with the higher rates (24-31%) observed among symptomatic populations, highlighting its rarity in community-based samples [24]. In the Kerala context, the prevalence in this study was lower than that reported in an earlier community-based study from Trivandrum, which found a prevalence of 23.5% among postmenopausal women [16]. The Trivandrum study incorporated direct clinical examinations and Pap smears, whereas our study relied on self-reported data from structured interviews with women aged 45-69 years. This methodological difference may account for underreporting and the absence of clinical validation, leading to lower prevalence estimates. In addition, Kerala’s relatively higher literacy rates, better health awareness, improved sanitation, and easier access to healthcare may contribute to the lower observed prevalence, reflecting behavioural and systemic factors that positively influence women’s health outcomes. The current study’s estimate of 5.5% for benign genital tract disorders, such as fibroids or atrophic changes, is also lower than figures reported in symptomatic populations [16].

With respect to treatment-seeking behaviour, all women with GM consulted a doctor; however, only 63.6% received treatment. This treatment gap, although narrower than many global and national trends, may be influenced by several factors, including financial constraints, cultural normalisation of symptoms, limited awareness about available treatment options, reliance on non-specialist care, and partial use of traditional medicine, which may not have been fully captured in the study data. Globally, systemic barriers often widen treatment gaps [25], while in India, financial and cultural factors further limit access to care [11]. In contrast, Kerala’s higher literacy rates and better healthcare infrastructure may contribute to higher consultation rates. The observed preference for private facilities (74.6%) and gynaecologists (67.3%) aligns with regional patterns, reflecting both accessibility and perceived quality of care.

The study’s findings contribute to the growing body of evidence on risk factors associated with GM among postmenopausal women. Women aged 45-59 years were significantly more likely to experience GM (AOR = 2.13), which may be attributed to the transition into the postmenopausal period, a stage characterised by hormonal fluctuations, declining oestrogen levels, and the onset of metabolic or endocrine disorders that increase susceptibility to conditions such as urogenital atrophy and pelvic organ prolapse. This finding is consistent with earlier Indian studies reporting a higher prevalence of GM among women in the early postmenopausal age group compared to those in older age brackets [4,23]. A strong association was also observed between GM and diabetes (AOR = 2.99), supporting international literature that recognises metabolic health as an important determinant of gynaecological outcomes. The association between thyroid disorders and GM (AOR = 4.21) is particularly noteworthy, aligning with emerging evidence that thyroid dysfunction and autoimmune processes may influence pelvic floor health [18,20]. Furthermore, menopause before the age of 47 was associated with a significantly increased risk of GM (AOR = 2.20), in line with previous findings that prolonged hypoestrogenic states predispose women to urogenital atrophy and pelvic organ prolapse [4,23]. Taken together, these findings underscore the critical role of age, endocrine function, and metabolic health in shaping gynaecological outcomes during the postmenopausal period and highlight the importance of improved risk stratification and proactive care strategies in diverse populations.

Strengths and limitations

This community-based study, which included both urban and rural populations, employed a multi-stage cluster sampling strategy and used a sample size calculated with a design effect to ensure representativeness. Examining both the prevalence of GM and treatment-seeking behaviour provides a more comprehensive understanding of postmenopausal women’s health needs.

However, the study has certain limitations. GMs were self-reported and not clinically validated, which may have led to underreporting or misclassification. The cross-sectional design restricts causal inference, and recall bias may have influenced responses. In addition, provider- and system-level barriers to care were not explored in depth, limiting insights into health system challenges.

## Conclusions

GM was found to be highly prevalent among postmenopausal women in Kerala, with significant associations observed for younger postmenopausal age, diabetes mellitus, thyroid disorders, and early menopause. Although healthcare-seeking was common, treatment uptake remained suboptimal, with a preference for private over public facilities. These findings highlight the need for actionable strategies. Integrating postmenopausal health into existing non-communicable disease clinics and reproductive health programmes at primary health centres may offer sustainable models for early detection and management. In addition, targeted health education campaigns delivered through community health workers (CHWs) could enhance awareness of symptoms and available treatment options, while structured referral linkages may strengthen continuity of care and reduce under-treatment.
